# Association Between Handgrip Strength and Suicidal Ideation: A Systematic Review and Meta‐Analysis

**DOI:** 10.1002/brb3.70647

**Published:** 2025-06-18

**Authors:** Prakasini Satapathy, Abhay M Gaidhane, Nasir Vadia, Soumya V Menon, Kattela Chennakesavulu, Rajashree Panigrahi, Manpreet Kaur, Ganesh Bushi, Muhammed Shabil, Diptismita Jena, Mayank Goyal, Harish Kumar, Anju Rani, Sanjit Sah, Khang Wen Goh

**Affiliations:** ^1^ Center for Global Health Research, Saveetha Medical College and Hospital, Saveetha Institute of Medical and Technical Sciences Saveetha University Chennai India; ^2^ Faculty of Data Science and Information Technology INTI International University Nilai Malaysia; ^3^ Jawaharlal Nehru Medical College, and Global Health Academy, School of Epidemiology and Public Health Datta Meghe Institute of Higher Education Wardha India; ^4^ Marwadi University Research Center, Department of Pharmaceutical Sciences, Faculty of Health Sciences Marwadi University Rajkot Gujarat India; ^5^ Department of Chemistry and Biochemistry, School of Sciences JAIN (Deemed to be University) Bangalore Karnataka India; ^6^ Department of Chemistry Sathyabama Institute of Science and Technology Chennai Tamil Nadu India; ^7^ Department of Microbiology, IMS and SUM Hospital Siksha ‘O’ Anusandhan (Deemed to be University) Bhubaneswar Odisha India; ^8^ Department of Pharmacy Chandigarh Pharmacy College, Chandigarh Group of Colleges‐Jhanjeri Mohali Punjab India; ^9^ Chitkara Centre for Research and Development Chitkara University Rajpura Punjab India; ^10^ School of Pharmaceutical Sciences Lovely Professional University Phagwara India; ^11^ University Center for Research and Development Chandigarh University Mohali Punjab India; ^12^ Medical Laboratories Techniques Department AL‐Mustaqbal University Hillah Babil Iraq; ^13^ Centre of Research Impact and Outcome Chitkara University Rajpura Punjab India; ^14^ Division of Research and Innovation Uttaranchal University Dehradun India; ^15^ IES Institute of Pharmacy IES University Bhopal Madhya Pradesh India; ^16^ New Delhi Institute of Management, Tughlakabad Institutional Area New Delhi India; ^17^ Department of Microbiology Graphic Era (Deemed to be University) Dehradun India; ^18^ SR Sanjeevani Hospital, Kalyanpur Siraha Nepal; ^19^ Department of Public Health Dentistry Dr. D. Y. Patil Medical College Hospital and Research Centre, Dr. D. Y. Patil Vidyapeeth (Deemed‐to‐be‐University) Pune Maharashtra India; ^20^ Department of Medicine Korea University Seoul South Korea; ^21^ Faculty of Mathematics and Natural Sciences Universitas Negeri Padang Padang Indonesia; ^22^ Graphic Era Hill University Clement Town Dehradun India

**Keywords:** Handgrip strength, suicidal ideation, mental health, systematic review, meta‐analysis

## Abstract

**Background:**

Suicidal ideation is a global public health concern, highlighting the need to identify modifiable risk factors. Handgrip strength (HGS), an objective measure of muscular strength, has been linked to mental health outcomes. This review synthesizes evidence on HGS and suicidal ideation, exploring modifiers such as sex.

**Methods:**

This systematic review and meta‐analysis, registered with PROSPERO and conducted in accordance with PRISMA guidelines, evaluated data retrieved from PubMed, Embase, and Web of Science up to November 30, 2024. The analysis focused on randomized controlled trials and observational studies—including case‐control, cohort, and cross‐sectional designs—that examined the relationship between HGS and suicidal ideation in human populations. A random‐effects model was employed to calculate pooled odds ratios (ORs) with 95% confidence intervals (CIs). Heterogeneity among studies was assessed using the *I* ^2^ statistic.

**Results:**

Out of 294 studies, 9 met the inclusion criteria for the systematic review, and 6 were eligible for meta‐analysis, involving 81,035 participants. The pooled analysis showed a nonsignificant association between HGS and suicidal ideation. For males, the pooled OR per 1 kg increase in HGS was 0.939 (95% CI, 0.875–1.009), and for females, it was 0.851 (95% CI, 0.662–1.094), indicating a potential but nonsignificant protective effect.

**Conclusion:**

This systematic review and meta‐analysis found no statistically significant association between handgrip strength and suicidal ideation in the pooled analysis. However, consistent trends observed in the qualitative synthesis suggest a potential relationship that warrants further investigation. Longitudinal studies are essential to elucidate the underlying mechanisms.

## Graphical abstract

Handgrip strength (HGS) and suicidal ideation were evaluated through a systematic review and meta‐analysis of nine studies including over 81,000 participants. While the pooled analysis showed no statistically significant association, qualitative trends suggested that lower HGS may increase suicidal ideation risk. Future longitudinal studies are needed to confirm HGS as a potential early marker for mental health vulnerability.

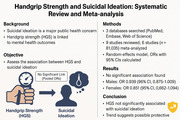

## 1 Introduction

Suicidal ideation is a major public health concern, with over 700,000 lives lost to suicide annually, according to the World Health Organization (WHO) (2024). Beyond these fatalities, millions engage in suicidal ideation or behaviors, highlighting the urgent need to identify modifiable risk factors for prevention (Turecki et al. 2019). Despite the global burden of suicide, efforts to reduce its prevalence have been limited by insufficient understanding of early warning signs and underlying mechanisms. Handgrip strength (HGS), a straightforward and objective measure of muscular strength, has recently emerged as a potential biomarker that may link physical and mental health outcomes (Soysal et al. 2021). Traditionally recognized for its predictive value in chronic disease, functional decline, and mortality, HGS has gained attention for its potential role in identifying individuals at risk for mental health conditions.

Emerging evidence suggests that lower HGS is associated with an increased risk of depression and anxiety, conditions often linked to suicidal ideation (Ganipineni et al. 2023). Hypothesized biological and psychosocial mechanisms may explain this relationship. Biologically, systemic inflammation and neuroendocrine dysfunction, such as dysregulation of the hypothalamic–pituitary–adrenal axis, may connect reduced muscular strength to mental health disorders by altering stress responses and neuroinflammatory processes (Tian et al. 2024). These pathways could impair psychological resilience, increasing vulnerability to suicidal ideation. Psychosocially, lower HGS is often associated with low self‐esteem, reduced functional independence, and perceived loss of autonomy, particularly in older adults or those with physical limitations (Chong et al. 2024). These factors may exacerbate psychological distress, contributing to suicidal ideation by diminishing one's sense of agency and social connectedness (Bağci Uzun et al. 2024). These biological and psychosocial mechanisms highlight HGS's potential as an accessible, noninvasive marker for identifying individuals at risk of mental health challenges, providing a theoretical foundation for exploring its association with suicidal ideation.

The association between HGS and suicidal ideation has gained research attention as part of the broader recognition of physical fitness as a holistic marker of health (Harner 2024). Evidence indicates that lower HGS correlates with greater psychological distress, including depressive symptoms and cognitive impairment (Noh and Park 2020). However, studies exploring the specific link between HGS and suicidal ideation have reported inconsistent findings, likely influenced by differences in study design, population characteristics, and methods of measuring HGS and suicidal ideation (Cao et al. 2020). Additionally, limited research has explored potential modifiers of this relationship, such as age, sex, and cultural or socioeconomic factors, further complicating the interpretation of existing data. Understanding the relationship between HGS and suicidal ideation is particularly important given the simplicity and accessibility of HGS measurement, which could enable widespread use in screening and preventive interventions.

This systematic review and meta‐analysis aim to consolidate and evaluate the evidence on the association between HGS and suicidal ideation. By pooling data from available studies, we seek to quantify the strength of this relationship and explore potential modifiers, including age and sex. Additionally, we assess the overall quality of the evidence and identify gaps in the literature that warrant further investigation. Clarifying this association could provide valuable insights into whether HGS serves as a meaningful marker for mental health and suicide risk, as well as inform future research and clinical practices focused on prevention. Understanding the potential role of HGS in mental health screening may pave the way for more comprehensive approaches to reducing the global burden of suicide.

## 2 Methods

This systematic review and meta‐analysis were prospectively registered with PROSPERO (Registration ID: CRD42024600561) to ensure transparency and methodological rigor. The review was conducted in accordance with the Preferred Reporting Items for Systematic Reviews and Meta‐Analyses (PRISMA) guidelines to promote methodological transparency and clarity (Table S1) (Page et al. 2021).

### 2.1 Eligibility Criteria

Eligibility criteria were established to ensure a focused and comprehensive review of the association between HGS and suicidal ideations. Studies were included if they involved human participants of any age and reported measures of both HGS and suicidal ideation, whether self‐reported, clinically assessed, or diagnosed. Eligible studies were limited to case‐control studies, randomized controlled trials, and observational designs, including cross‐sectional and cohort studies, that evaluated HGS as an indicator of physical strength. Only published articles in English available through November 2024 were considered. Exclusion criteria encompassed studies focusing on death by suicide, animal subjects, qualitative research, reviews, discussion papers, editorials, commentaries, case reports, letters to the editor, and abstract‐only publications without full‐text availability. This approach ensured the inclusion of high‐quality studies directly addressing the research question while minimizing potential bias from irrelevant or incomplete data sources (Table S2).

### 2.2 Search Strategy

A comprehensive search strategy was implemented across PubMed, Embase, and Web of Science to identify studies examining the association between HGS and suicidal ideation. Search terms included combinations of MeSH or Emtree terms and free‐text keywords, such as “Handgrip Strength,” “Muscle Strength,” “Grip Strength,” “Suicidal Ideation,” “Suicidal Thoughts,” and “Suicidal Behavior,” tailored to each database. Boolean operators and truncations were applied to maximize the retrieval of relevant articles. In PubMed, the search included terms such as (“Muscle Strength”[Mesh] OR “Hand Strength”[Mesh]) AND (“Suicidal Ideation”[Mesh] OR “Suicidal Thoughts”), while Embase used terms such as (“handgrip strength”/exp OR “muscle strength”/exp) AND (“suicidal ideation”/exp OR “suicidal thoughts”/exp). Searches were conducted through November 30, 2024, and were restricted to English‐language publications. Full search strategies and exact terms used for each database are provided in Table S3.

### 2.3 Screening and Data Extraction

Screening was systematically carried out by two independent reviewers using Nested Knowledge software. After removing duplicate records, the titles and abstracts of all identified studies were examined to determine potential eligibility. Full‐text articles of the shortlisted studies were then reviewed for final inclusion based on predefined eligibility criteria. Disagreements between reviewers were resolved through discussion or, if needed, consultation with a third reviewer. Data extraction was conducted using a structured Excel sheet, capturing key information such as study characteristics, participant demographics, methods of HGS assessment, tools used to evaluate suicidal ideation, confounding factors, and effect size estimates (e.g., odds ratios [ORs], hazard ratios, or correlation coefficients). Extracted data were thoroughly cross‐checked for accuracy and consistency.

### 2.4 Quality Assessment

The quality of included studies was evaluated using the Newcastle–Ottawa Scale (NOS) (Padhi et al. 2024), which assesses methodological quality across three domains: participant selection, comparability of groups, and outcome assessment, with a maximum score of 9. Studies scoring 7 or higher were classified as high quality, those scoring 4–6 as moderate quality, and those scoring 0–3 as low quality. Two reviewers independently assessed and scored the studies, resolving any disagreements through discussion.

### 2.5 Evidence Synthesis

All statistical analyses were conducted using R software version 4.4 (Wang 2023). To address variability across studies, a random‐effects model was employed for the meta‐analysis, with pooled effect sizes presented as ORs and their 95% confidence intervals to assess the relationship between HGS and suicidal ideation. Study heterogeneity was measured using the *I* ^2^ statistic, with values above 50% indicating notable heterogeneity (Langan et al. 2019, Gandhi et al. 2023). Publication bias was evaluated visually through a Doi plot and quantitatively using the LFK index (Furuya‐Kanamori et al. 2018, Shamim 2023). Subgroup analyses were performed based on predetermined variables, including age and sex, to explore potential contributors to heterogeneity.

## 3 Results

### 3.1 Literature Search

**FIGURE 1:**
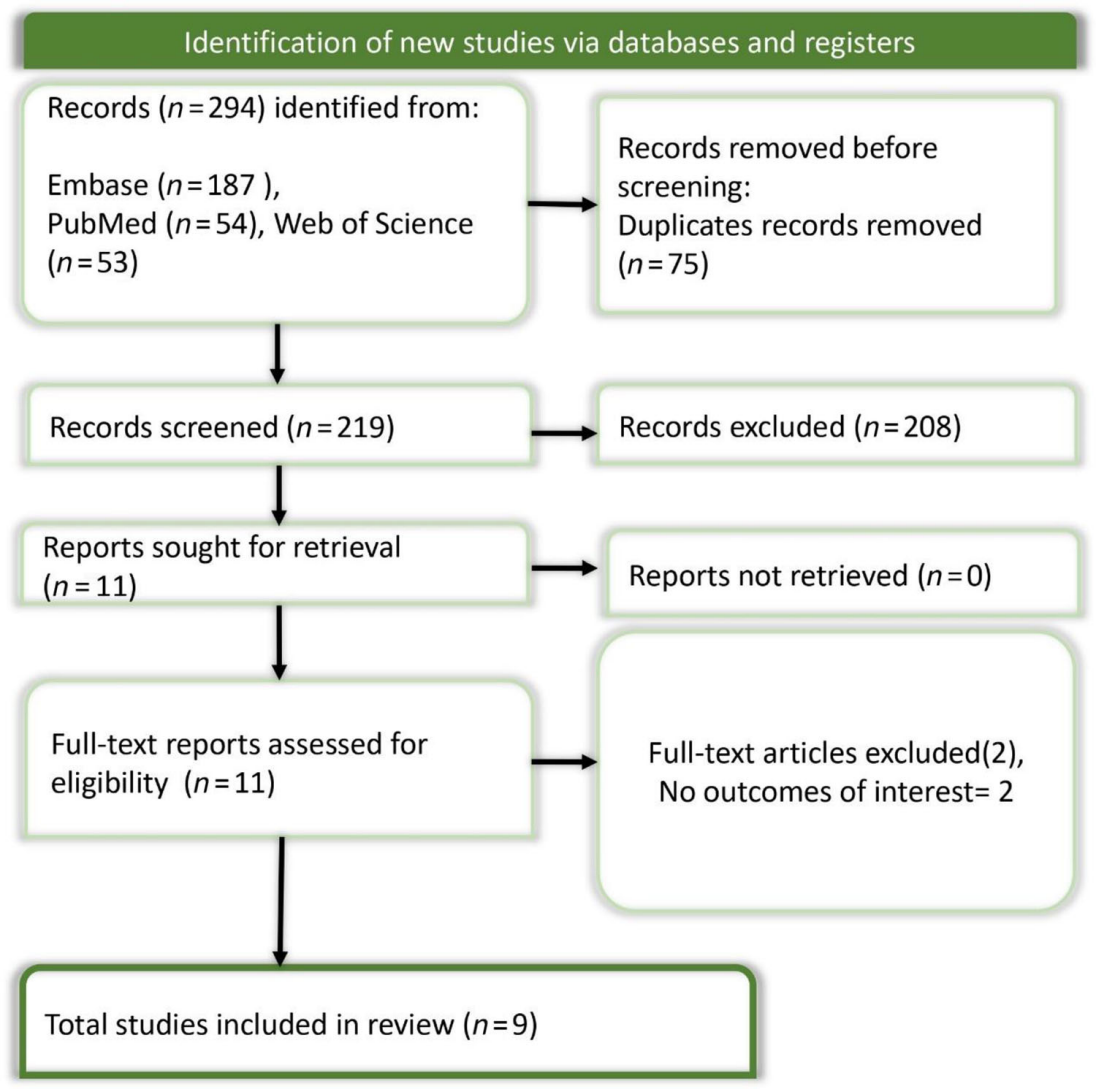
PRISMA flow chart showing the study selection process.

The literature search identified 294 records from three databases: Embase (*n* = 187), PubMed (*n* = 54), and Web of Science (*n* = 53). After removing 75 duplicates, 219 unique records were screened by title and abstract. Of these, 208 were excluded for not meeting eligibility criteria. Eleven full‐text articles were assessed, with two excluded due to irrelevant outcomes. Ultimately, nine studies were included in the systematic review, and six were eligible for meta‐analysis. The PRISMA flow diagram (Figure 1) outlines the study selection process.

### 3.2 Summary Characteristics of Studies

The nine studies included in this systematic review comprised a total sample size of 81,035 participants, representing diverse populations and study designs. Seven studies were cross‐sectional, while two were cohort studies, conducted across various countries, including six studies from Korea (Hwang and Ahn 2021, Hwang, Ahn, and Choi 2021, Noh and Park 2020, Kwak and Kim 2022, Han et al. 2019, Lee 2018), one from the United States (Cao et al. 2020), one from Russia (Bikbov et al. 2023), and one involving low‐ and middle‐income countries (China, Ghana, India, Mexico, Russia, and South Africa) (Smith et al. 2024). Participant ages ranged from adolescents to older adults, with male representation varying between 43.7% and 52.5%. HGS was assessed using validated methods, such as digital dynamometers and Smedley's hand dynamometer, with measurements expressed as either absolute strength in kilograms or relative strength. Suicidal ideation was evaluated through validated questionnaires or self‐reported measures, with phrasing slightly differing across studies (e.g., “Have you ever seriously thought about committing suicide in the last 12 months?” or “Did you think of death, or wish you were dead?”). This variability in population demographics and methodologies reflects the broad scope of the included studies while also highlighting differences in HGS and suicidal ideation assessments across cultural and clinical contexts (Table 1). The quality of the included studies was assessed using the NOS, with results, including detailed scores, presented in Table S4.

**TABLE 1:** Summary characteristics of included studies.

**Study**	**Study design**	**Country**	**Male %**	**Mean age**	**Handgrip diagnosis**	**Total sample size**	**NOS score**
Hwang and Ahn (2021)	Cross‐sectional study	South Korea	44.7	49.8	Digital grip strength dynamometer	14325	6
Hwang, Ahn, and Choi (2021)	Cross‐sectional study	South Korea	52.5	14.9	Digital grip strength dynamometer	3530	6
Noh and Park (2020)	Retrospective cohort study	South Korea	45.58	Above 65	NA	2652	6
Kwak and Kim (2022)	Cross‐sectional study	South Korea	NA	NA	Digital grip strength dynamometer	1254	6
Cao et al. (2020)	Cross‐sectional study	US	49.97	47.4	HGS in kilograms (kg) was defined as the maximum value from the dominant hand	8903	6
Han et al. (2019)	Cross‐sectional study	South Korea	45.8	69.55	NA	3169	9
Bikbov et al. (2023)	Prospective cohort study	Russia	43.7	59	HGS in kilograms (kg) was defined as the maximum value from the dominant hand	5893	4
Lee (2018)	Cross‐sectional study	South Korea	51.84	50.9	Digital grip strength dynamometer	4180	6
Smith et al. (2024)	Cross‐sectional study	Low and middle‐income countries (China, Ghana, India, Mexico, Russia, and South Africa)	47.9	62.4	Smedley's hand dynamometer	34,129	9

### 3.3 Meta‐Analysis

#### 3.3.1 Gender‐Specific Association Between Per 1 Kg Increase in Grip Strength and Suicidal Ideation

**FIGURE 2:**
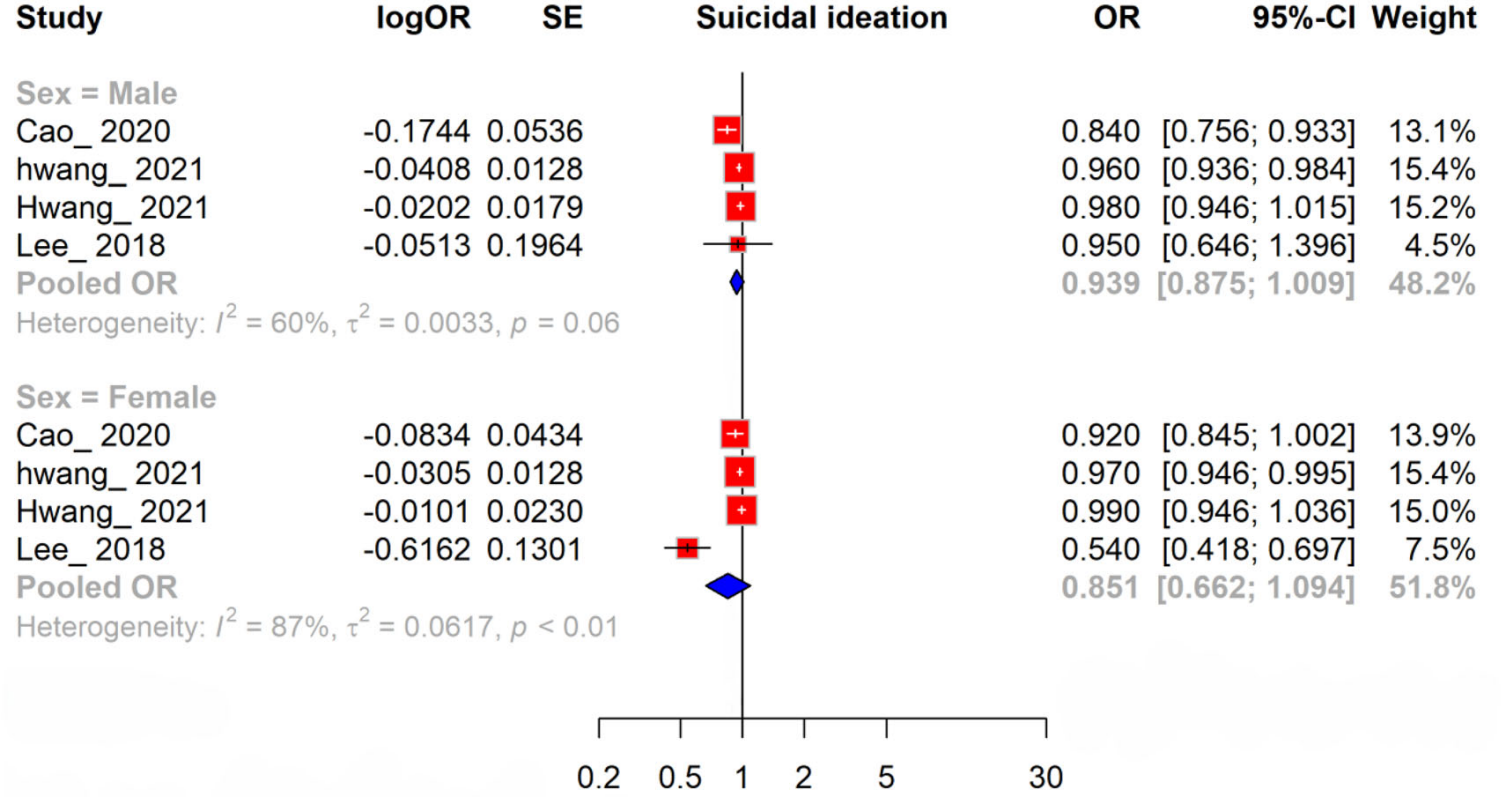
Forest plot illustrating the association between per 1 kg increase in grip strength and suicidal ideation.

Among four studies, the association between HGS and suicidal ideation was analyzed separately for males and females, expressed as ORs per 1 kg increase in grip strength. For males, the pooled OR was 0.939 (95% CI, 0.875–1.009), indicating a nonsignificant association with moderate heterogeneity (*I* ^2^ = 60%). For females, the pooled OR was 0.851 (95% CI, 0.662–1.094), suggesting a stronger but still nonsignificant association, with high heterogeneity (*I* ^2^ = 87%). These findings indicate that while increased HGS may have a potential protective effect against suicidal ideation, the associations were not statistically significant. Notably, the trend appeared more pronounced among females, suggesting a possible sex‐specific difference in the relationship between HGS and suicidal ideation. This trend, though not significant, highlights the potential clinical relevance of HGS as a simple, noninvasive marker for identifying individuals at risk of suicidal ideation, particularly in settings where mental health screening resources are limited. However, the variability across studies, as reflected by the heterogeneity, necessitates cautious interpretation and further research to validate these observations (Figure 2).

#### 3.3.2 Pooled Analysis of the Association Between HGS and Suicidal Ideation

**FIGURE 3:**
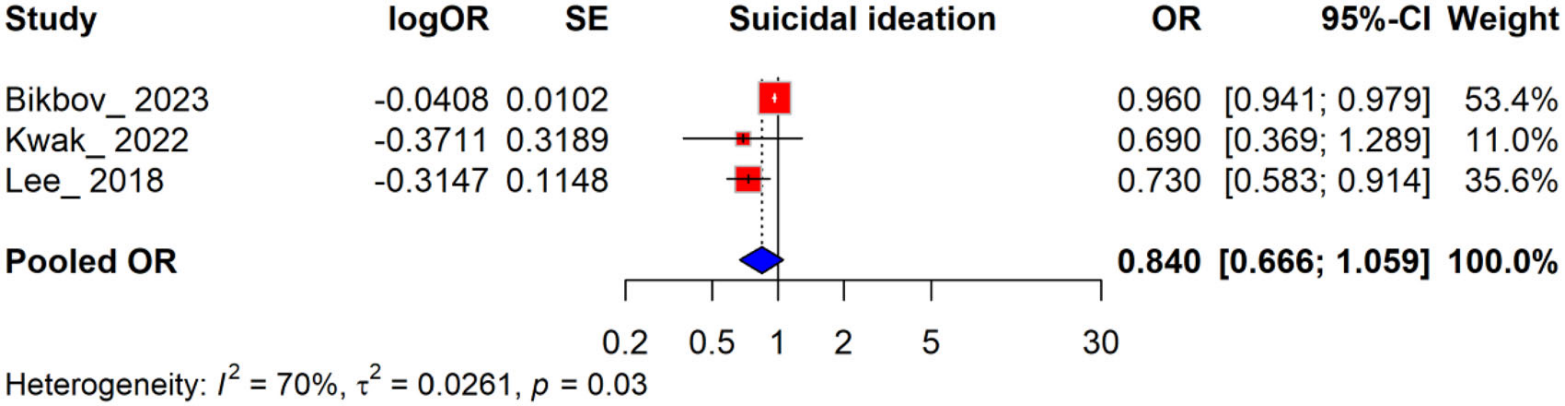
Forest plot illustrating the association between handgrip strength and suicidal ideation: pooled analysis.

The association between HGS and suicidal ideation was assessed across three studies, with a pooled OR of 0.840 (95% CI, 0.666–1.059), indicating a nonsignificant association between increasing HGS and suicidal ideation. Individual study ORs ranged from 0.690 to 0.960, with weight contributions varying by sample size and precision (e.g., Bikbov_2023 contributed 53.4% of the weight). Heterogeneity was moderate (*I* ^2^ = 70%), suggesting variability among studies. While the findings suggest a potential protective effect of greater HGS on suicidal ideation, the high heterogeneity emphasizes the need for cautious interpretation and further research to validate these results (Figure 3).

### 3.4 Qualitative Synthesis

A qualitative synthesis of three studies was carried out to assess the relationship between HGS and suicidal ideation, as differences in study designs and reporting methods prevented their inclusion in the meta‐analysis. Despite these variations, all three studies identified a significant association, indicating that lower HGS is linked to a higher likelihood of suicidal ideation.

The first study, conducted in Korea by Noh and Park (2020), found that a 1 kg decrease in HGS was associated with a 7% increase in the odds of suicidal ideation in males (OR = 1.07; 95% CI, 1.02–1.12), while the odds for females were not significant (OR = 0.99; 95% CI, 0.94–1.05). These results remained consistent after adjusting for various confounding factors, including age, marital status, education level, employment, multimorbidity, household income, residence, body mass index, smoking, alcohol consumption, physical activity, and waist circumference.

The second study, conducted in Korea by Han et al. (2019), categorized HGS into tertiles based on sex. For women, the tertiles were defined as Tertile I (HGS < 19.9 kg), Tertile II (19.9 kg ≤ HGS < 24.0 kg), and Tertile III (HGS ≥ 24.0 kg). For men, the tertiles were Tertile I (HGS < 32.9 kg), Tertile II (32.9 kg ≤ HGS < 38.8 kg), and Tertile III (HGS ≥ 38.8 kg). The study reported that individuals in the lowest tertile of HGS had a significantly higher prevalence of suicidal ideation compared to those in the highest tertile (OR = 1.85; 95% CI, 1.31–2.62). This relationship remained significant after controlling for confounders, emphasizing the potential of HGS as a marker for suicidal ideation.

The third study, conducted in low‐ and middle‐income countries by Smith et al. (2024), demonstrated a clear dose–response relationship between decreasing HGS and increasing odds of suicidal ideation. Participants were divided into quintiles based on HGS levels, with males in the lowest quintile (Q5, ≤ 21 kg) and females in the lowest quintile (Q5, ≤ 12 kg) showing the highest risk. For overall suicidal ideation, adjusted odds ratios (aORs) increased progressively with decreasing HGS: Q2 = 2.15 (95% CI, 1.05–4.39), Q3 = 2.78 (95% CI, 1.06–7.32), Q4 = 3.53 (95% CI, 1.68–7.42), and Q5 = 6.79 (95% CI, 2.80–16.48), compared to the highest quintile (Q1, reference = 1). When analyzed by sex, similar trends were observed. For males, those in the lowest quintile had an aOR of 5.83 (95% CI, 2.19–15.50), while females in the lowest quintile had an aOR of 6.11 (95% CI, 2.27–16.46), both significantly higher than their respective highest quintiles. These findings strongly indicate that as HGS decreases, the likelihood of suicidal ideation markedly increases, underscoring the critical link between physical strength and mental health vulnerability in both sexes (Table 2). The relationship between HGS and suicidal ideation is consistent across diverse cultural and demographic contexts, emphasizing the potential of HGS as a global marker for mental health risks. Collectively, these findings provide consistent evidence of a significant inverse association between HGS and suicidal ideation across diverse populations and settings.

**TABLE 2:** Study Characteristics and Adjusted Effect Sizes for Handgrip Strength and Suicidal Ideation.

**Study**	**Handgrip strength**	**Effect size [OR (95% CI)]**			**Adjusted factors**
		**Male**	**Female**	**Overall**	
Hwang and Ahn (2021)	Digital grip strength dynamometer	0.96 (0.94–0.99)	0.97 (0.95–1.00)	NA	Age, education, marital status, economic status, smoking, physical activity, obesity, comorbidities, depressive mood
Hwang, Ahn, and Choi (2021)	Digital grip strength dynamometer	0.98 (0.95–1.02)	0.99 (0.95–1.04)	NA	Age, household income, and body mass index
Noh and Park (2020)	NA	1.07 (1.02–1.12)	0.99 (0.94–1.05)	NA	Age, marital status, education level, employment, household income, residence, multimorbidity, smoking, alcohol intake, physical activity, body mass index, waist circumferences
Kwak and Kim (2022)	Digital grip strength dynamometer	NA	NA	0.69 (0.31–1.56)	Race, ethnicity, education, marital status, smoking status, BMI)
Cao et al. (2020)	Handgrip strength in kilograms (kg) was defined as the maximum value from the dominant hand	0.84 (0.74–0.95)	0.92 (0.9–1.07)	NA	Race, ethnicity, education, marital status, smoking status, BMI)
Han et al. (2019)	NA	NA	NA	I (5 ≤ PHQ‐9 ≤ 9) = 1.85 (1.31–2.62) II (10 ≤ PHQ‐9 ≤ 14) = 1.50 (1.07–2.11) III (PHQ‐9 ≥ 15) = 1	All sociodemographic and health‐related variables
Bikbov et al. (2023)	Handgrip strength in kilograms (kg) was defined as the maximum value from the dominant hand	NA	NA	0.96 (0.94–0.98)	NA
Lee (2018)	Digital grip strength dynamometer	0.95 (0.63–1.4)	0.54 (0.34–0.85)	0.73 (0.54–0.99)	All independent factors, including depressive mood
Smith et al. (2024)	Smedley's hand dynamometer	Q1 (highest): 1 Q2: 1.62 (0.60–4.38) Q3: 1.14 (0.42–3.04) Q4: 2.75 (1.05–7.21) Q5 (lowest): 5.83 (2.19–15.50)	Q1 (highest): 1 Q2: 2.13 (0.92–4.93) Q3: 3.37 (1.10–10.35) Q4: 3.56 (1.49–8.52) Q5 (lowest): 6.11 (2.27–16.46)	Q1 (highest): 1 Q2: 2.15 (1.05–4.39) Q3: 2.78 (1.06–7.32) Q4: 3.53 (1.68–0.42) Q5 (lowest): 6.79 (2.80–16.48)	All sociodemographic and health‐related variables

### 3.5 Publication Bias

**FIGURE 4:**
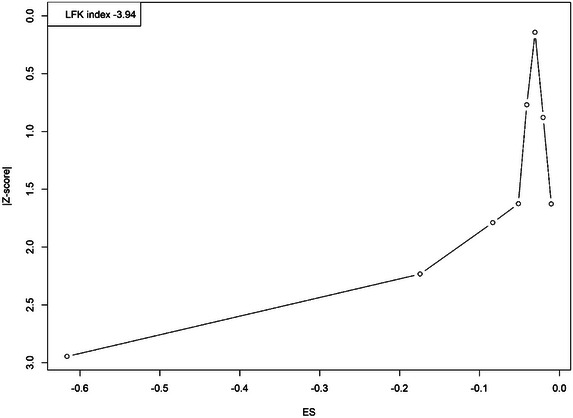
Doi plots depicting the publication bias.

Publication bias was evaluated using a Doi plot and the LFK index. The Doi plot showed asymmetry, with the majority of studies clustering on one side, indicating potential bias in the available data. The LFK index was calculated as −3.94, reflecting significant asymmetry and a considerable risk of publication bias. This asymmetry suggests that smaller studies with nonsignificant results may be underrepresented in the analysis. The presence of publication bias underscores the need for careful interpretation of these findings and highlights the importance of future research to address this limitation by incorporating a wider range of evidence, including unpublished or negative results (Figure 4).

## 4 Discussion

This systematic review and meta‐analysis is the first of its kind to evaluate the association between HGS and suicidal ideation, consolidating data from nine studies encompassing over 81,000 participants across diverse populations. The findings provide valuable insights into the relationship between HGS and mental health. While the quantitative analysis indicated nonsignificant results, suggesting no clear relationship between HGS and suicidal ideation, the qualitative analysis demonstrated that lower HGS is associated with a higher likelihood of suicidal ideation. However, substantial heterogeneity, publication bias, and limitations in study designs warrant cautious interpretation of these findings and highlight areas for future research.

The pooled analysis showed no significant association between HGS and suicidal ideation, with a pooled OR of 0.840 (95% CI, 0.666–1.059). Subgroup analyses by sex revealed a pooled OR of 0.939 (95% CI, 0.875–1.009) for males, with moderate heterogeneity (*I* ^2^ = 60%), and 0.851 (95% CI, 0.662–1.094) for females, with high heterogeneity (*I* ^2^ = 87%), suggesting a stronger but nonsignificant protective trend in females. The observed heterogeneity (*I* ^2^ = 60%–91%) likely stems from methodological and population differences. For instance, the predominance of cross‐sectional studies (seven of nine) versus cohort studies (two) may contribute to inconsistent findings, as cross‐sectional designs limit causal inference. Variability in HGS measurement techniques, such as digital dynamometers (e.g., Hwang, Ahn, and Choi 2021; Lee 2018) versus Smedley's hand dynamometer (e.g., Smith et al. 2024), could introduce discrepancies in pooled estimates. Participant characteristics, including age (ranging from adolescents to older adults), sex, and cultural differences, also likely contribute, as mental health stigma and physical activity levels vary across populations. The predominance of Korean studies (six of nine) may reduce generalizability, potentially increasing heterogeneity when compared to studies from the United States, Russia, or low‐ and middle‐income countries. Furthermore, differences in suicidal ideation questionnaire phrasing (e.g., “Have you ever seriously ideation about committing suicide in the last 12 months?” vs. “Did you think of death, or wish you were dead?”) and varying confounder adjustments (e.g., age, socioeconomic status, comorbidities) across studies may exacerbate variability. These factors underscore the need for standardized methodologies in future research to minimize heterogeneity and enhance reliability.

The relationship between HGS and suicidal ideation may vary across age groups, regions, and socioeconomic status, potentially influencing the observed trends. Older adults with lower HGS may experience greater physical limitations and psychological distress, increasing their risk of suicidal ideation, as evidenced by Smith et al. (2024), which reported a dose–response relationship in adults aged ≥ 50 years from low‐ and middle‐income countries. Regionally, the predominance of Korean studies (six of nine) may reflect cultural factors, such as high mental health stigma or widespread physical fitness practices, which could influence the HGS‐suicidal ideation association, while distinct findings from low‐ and middle‐income countries (Smith et al. 2024) suggest that limited healthcare access may exacerbate this relationship. Lower socioeconomic status, often associated with reduced physical activity and poorer health outcomes, may further intensify the link between low HGS and suicidal ideation, as supported by Han et al. (2019), who found significant associations in community‐dwelling older adults with varying socioeconomic backgrounds. Due to limited data, subgroup analyses by age, region, or socioeconomic status were not feasible in this review.

The discrepancies between the nonsignificant meta‐analysis results and the significant findings in the qualitative synthesis warrant further exploration. The meta‐analysis, which included six studies, yielded a nonsignificant pooled OR (0.840, 95% CI, 0.666–1.059), likely influenced by high heterogeneity (*I* ^2^ = 70%), publication bias (LFK index = −3.94), and the limited number of studies, which may have reduced statistical power. In contrast, the qualitative synthesis of three studies (Noh and Park 2020; Han et al. 2019; Smith et al. 2024) identified significant associations, with Noh and Park (2020) reporting a 7% increase in odds of suicidal ideation per 1 kg decrease in HGS in Korean males (OR = 1.07; 95% CI, 1.02–1.12), Han et al. (2019) finding higher odds in the lowest HGS tertile (OR = 1.85; 95% CI, 1.31–2.62), and Smith et al. (2024) demonstrating a dose–response relationship in low‐ and middle‐income countries (e.g., aOR = 6.79; 95% CI, 2.80–16.48 for the lowest quintile). These significant findings may reflect the qualitative studies’ ability to capture specific population effects or nuanced relationships, such as dose–response patterns, that are diluted in pooled analyses. Potential reasons for these discrepancies include differences in study designs, with cross‐sectional studies (predominant in the meta‐analysis) limiting causal inference and potentially masking true associations. Variations in HGS measurement methods (e.g., digital vs. Smedley's dynamometers) and inconsistent confounder adjustments (e.g., socioeconomic status, comorbidities) across studies may further contribute to conflicting findings. Future research should employ longitudinal designs and standardized methodologies to resolve these discrepancies and validate the observed trends.

Despite the nonsignificant pooled results, the qualitative synthesis and subgroup trends suggest that HGS may serve as a proxy for physical and psychological resilience. Studies such as Noh and Park (2020) reported a 7% increase in odds of suicidal ideation per 1 kg decrease in HGS in males (OR = 1.07; 95% CI, 1.02–1.12), while Han et al. (2019) found higher odds in the lowest HGS tertile (OR = 1.85; 95% CI, 1.31–2.62). Smith et al. (2024) demonstrated a dose–response relationship in low‐ and middle‐income countries, with odds of suicidal ideation increasing across quintiles of decreasing HGS. These findings highlight the clinical relevance of HGS as a cost‐effective, noninvasive marker for identifying individuals at risk of suicidal ideation, particularly in resource‐limited settings. Integrating HGS measurements into routine health assessments could provide an early indicator of mental health vulnerability, complementing psychological screening methods.

The simplicity and low cost of HGS measurement further support its potential as a screening tool across diverse settings. The suggestive trends observed suggest that lower HGS may reflect underlying physical or psychological factors, such as reduced functional independence or self‐esteem, which could contribute to suicidal ideation. These insights emphasize the need for longitudinal studies to explore causality and mechanisms, such as systemic inflammation or neuroendocrine dysfunction, linking HGS to mental health outcomes. Clinically, incorporating HGS assessments into primary care or community health programs could facilitate early identification of at‐risk individuals, especially in populations with limited access to mental health resources.

Our findings align with prior research highlighting HGS as a marker of both physical and mental health. Zasadzka et al. (2021) demonstrated a weak but significant negative correlation between HGS and depressive symptoms (*r* = −0.148; 95% CI, −0.206 to −0.091), highlighting that low muscle strength intensifies depressive symptoms in older adults and should prompt clinical screening for depression, a condition often underdiagnosed in this population. Similarly, Soysal et al. (2021) conducted an umbrella review summarizing systematic reviews and meta‐analyses, consistently linking low HGS with adverse health outcomes, including depression and anxiety, and positioning HGS as a marker of both physical and psychological well‐being. Zhang et al. (2022) extended these findings by showing that weaker HGS was significantly associated with higher depression scores in cancer survivors, underscoring the heightened vulnerability of this group. While these studies primarily focus on depression, our study expands the scope by exploring the association between HGS and suicidal ideation, a critical yet less‐studied mental health outcome. Bellón et al. (2024), in a cross‐sectional study from the AGUEDA trial, further complemented this evidence by demonstrating that HGS positively correlated with self‐esteem (*β* = 0.558; 95% CI, 0.168–0.949), while perceived strength was negatively associated with depressive symptoms (*β* = −0.271; 95% CI, −0.491 to −0.049), reinforcing the multidimensional role of HGS in mental health. Zhang et al. (2021) added to this understanding by examining low HGS as a predictor of 90‐day mortality among older Chinese inpatients, showing its association with poorer physical and mental health outcomes, emphasizing its broad utility as a health indicator. Collectively, these studies and our findings underscore that low HGS is not just a marker of physical decline but also a significant indicator of mental health vulnerability. This evidence highlights the need for further research to explore the underlying mechanisms linking muscular strength to mental health outcomes, including neuroendocrine dysfunction and systemic inflammation, and to evaluate the role of HGS as a simple, noninvasive tool for identifying individuals at risk for severe mental health challenges, including suicidal ideation.

Despite its strengths, this study has several limitations. Substantial heterogeneity was observed across studies (*I* ^2^ = 60%–91%), reflecting variability in study designs, populations, and methods of assessing HGS and suicidal ideation. While random‐effects models accounted for some variability, residual heterogeneity may have influenced the pooled estimates. Publication bias was evident, as indicated by the asymmetry in the Doi plot and the LFK index (−3.94), suggesting that smaller studies with nonsignificant results may be underrepresented, potentially overestimating the association between HGS and suicidal ideation. The small number of included studies further limits the robustness of these findings. Additionally, the cross‐sectional design of most studies precludes causal inference, as reverse causation remains possible, with mental health conditions contributing to reduced physical activity and muscle strength. Longitudinal studies are needed to clarify the temporal relationship between HGS and suicidal ideation. The lack of studies from diverse countries also limits generalizability, as regional and cultural factors may influence both HGS and mental health outcomes. Future research should incorporate standardized and validated tools, expand representation across diverse populations, and include longitudinal designs to improve the reliability and applicability of the findings.

To advance understanding of the relationship between HGS and suicidal ideation, future research should address the identified limitations. Longitudinal studies with diverse populations are essential to establish causality and explore potential mediators and moderators, such as systemic inflammation, physical activity, and psychosocial factors. The inclusion of larger and more representative samples, particularly from underrepresented regions, will enhance the generalizability of findings, while sex‐specific and age‐specific analyses should be prioritized to identify vulnerable subgroups and develop tailored interventions. Additionally, integrating HGS measurement into routine clinical practice could provide valuable data for screening and risk stratification, especially in low‐resource settings where its simplicity and low cost make it an attractive mental health screening tool.

## 5 Conclusion

This systematic review and meta‐analysis found no statistically significant association between HGS and suicidal ideation in the pooled analysis. However, consistent trends observed in the qualitative synthesis suggest a potential relationship that warrants further investigation. Longitudinal studies are essential to elucidate the underlying mechanisms.

## Author Contributions

**Prakasini Satapathy**: conceptualization, investigation, writing – original draft, writing – review and editing. **Abhay M Gaidhane;** software, conceptualization, methodology, writing – review & editing, resources. **Nasir Vadia**: methodology, conceptualization, writing – review and editing, project administration. **Soumya V Menon**: methodology, conceptualization, writing – original draft, project administration, software. **Kattela Chennakesavulu**: methodology, conceptualization, writing – review and editing, project administration. **Rajashree Panigrahi**: data curation, writing – original draft, conceptualization, writing – review and editing. **Manpreet Kaur**: data curation, writing – review and editing, conceptualization, methodology, software, resources. **Ganesh Bushi**: writing – original draft, conceptualization, writing – review and editing, methodology. **Muhammed Shabil**: methodology, conceptualization, writing – review and editing. **Diptismita Jena**: conceptualization. **Mayank Goyal**: conceptualization, methodology, writing – review and editing. **Harish Kumar**: conceptualization, formal analysis, software, writing – review and editing, methodology. **Anju Rani**: conceptualization, writing – original draft, methodology, writing – review and editing. **Sanjit Sah**: writing – review and editing, methodology, conceptualization. **Khang Wen Goh**: conceptualization, methodology, software, data curation.

## Conflicts of Interest

The authors declare no conflicts of interest.

## Peer Review

The peer review history for this article is available at https://publons.com/publon/10.1002/brb3.70647

## Ethics Statement

The authors have nothing to report.

## Supporting information




**Supporting Table 1**: PRISMA Checklist
**Supporting Table 2**: Inclusion and Exclusion Criteria
**Supporting Table 3**: The adjusted search term as per the searched electronic database
**Supporting Table 4**: NOS for the quality assessment of included studies.

## Data Availability

All data generated or analyzed during this study are included in this published article (and its supporting information files).
